# A boy with conduct disorder (CD), attention deficit hyperactivity disorder (ADHD), borderline intellectual disability, and 47,XXY syndrome in combination with a 7q11.23 duplication, 11p15.5 deletion, and 20q13.33 deletion

**DOI:** 10.1186/s13034-016-0121-8

**Published:** 2016-09-15

**Authors:** Gerasimos Kolaitis, Christian G. Bouwkamp, Alexia Papakonstantinou, Ioanna Otheiti, Maria Belivanaki, Styliani Haritaki, Terpsihori Korpa, Zinovia Albani, Elena Terzioglou, Polyxeni Apostola, Aggeliki Skamnaki, Athena Xaidara, Konstantina Kosma, Sophia Kitsiou-Tzeli, Maria Tzetis

**Affiliations:** 1 Department of Child Psychiatry, Medical School, National and Kapodistrian University of Athens, “Aghia Sophia” Children’s Hospital, 11527 Athens, Greece; 2 1st Department of Pediatrics, Medical School, National and Kapodistrian University of Athens, “Aghia Sophia” Children’s Hospital, 11527 Athens, Greece; 3 Department of Medical Genetics, Medical School, National and Kapodistrian University of Athens, Athens, 11527 Greece; 4 Department of Child and Adolescent Psychiatry/Psychology, Erasmus University Medical Center, 3015 CN Rotterdam, The Netherlands; 5 Department of Psychiatry and Department of Clinical Genetics, Erasmus University Medical Center, 3015 CN Rotterdam, The Netherlands

**Keywords:** 47,XYY syndrome, 7q11.23 Williams–Beuren syndrome region micro duplication, Conduct disorder, ADHD, ASD, 20q13.33 deletion syndrome, 11p15.5 deletion

## Abstract

**Background:**

This is a case with multiple chromosomal aberrations which are likely etiological for the observed psychiatric phenotype consisting of attention deficit hyperactivity and conduct disorders.

**Case presentation:**

We report on an 11 year-old boy, admitted to the pediatric hospital for behavioral difficulties and a delayed neurodevelopmental trajectory. A cytogenetic analysis and high-resolution microarray comparative genomic hybridization (CGH) analysis was performed. The cytogenetic analysis revealed 47,XYY syndrome, while CGH analysis revealed an additional duplication and two deletions. The 7q11.23 duplication is associated with speech and language delay and behavioral symptoms, a 20q13.33 deletion is associated with autism and early onset schizophrenia and the 11p15.5 microdeletion is associated with developmental delay, autism, and epilepsy. The patient underwent a psychiatric history, physical examination, laboratory testing, and a detailed cognitive, psychiatric, and occupational therapy evaluation which are reported here in detail.

**Conclusions:**

In the case of psychiatric patients presenting with complex genetic aberrations and additional psychosocial problems, traditional psychiatric and psychological approaches can lead to significantly improved functioning. Genetic diagnostic testing can be highly informative in the diagnostic process and may be applied to patients in psychiatry in case of complex clinical presentations.

## Background

With the advent of novel genetic technologies, it has become accessible to genetically assess patients with complex clinical presentations. Historically in psychiatry, genetic assessment is not part of the standard diagnostic procedure. However, several recent papers have described a considerable diagnostic yield when looking for structural genetic variants (deletions, duplications, and larger chromosomal aberrations) [1, 2].

47,XYY syndrome is such a chromosomal abnormality known to occur in 1 out of 1000 male births [3]. The etiology of the additional Y chromosome is a paternal nondisjunction at meiosis II. In some cases, the failure occurs in the cell division of the postzygotic mitosis in early embryonic development and produces a mosaic 46,XY/47,XYY karyotype. The chromosomal aberration was first discovered and diagnosed by Sandberg and colleagues in the US [4] and is also originally known as Jacob’s syndrome [5]. 47,XYY syndrome is difficult to diagnose since there is no hallmark phenotype by which the syndrome can be recognized; the only phenotype often associated with the 47,XYY karyotype is that of tall stature. Furthermore, given that paternal nondisjunction lies at the basis of the etiology of the genotype, 47,XYY syndrome is not known to be hereditary, but instead occurs de novo.

Although 47,XYY syndrome was first described in 1961, an increased risk for neurodevelopmental disorders has been documented only recently [6]. A child with this abnormality may develop mild learning disability, delayed speech and language development, reading difficulties needing remedial help [7, 8] and motor difficulties [9]. Families frequently report behavioral problems such as temper tantrums, impulsiveness and aggressive or violent behavior. Furthermore, they report their sons are not empathetic and that they lack social skills [3, 10]. These boys are described as restless, hyperactive, or inattentive while the rates of anxiety and depression are above those for the general population [9, 11]. An association has been found between the 47,XYY genotype and autism spectrum disorders (ASD) [3, 10, 12]. In a cross-sectional descriptive study of males with 47,XYY syndrome, there was found an increased incidence of asthma, seizures, tremor, and ASD compared to the general population rates. Prenatally diagnosed boys scored significantly better on cognitive testing and were less likely to be diagnosed with ASD [13].

Duplications of 7q11.23, the locus corresponding to the deletion in Williams–Beuren Syndrome (WBS), have been implicated in ASD [14–18]. The common denominator in patients with the 7q11.23 duplication appears to be the neurodevelopmental delay, speech delay, separation anxiety disorders, and obsessive–compulsive behavior experienced in childhood and early adolescence.

The aim of the present case report is three-fold: (1) to bring under the attention that genetic syndromes may underlie psychiatric disorders, (2) to present this case with multiple chromosomal aberrations which are probably etiological for the observed psychiatric phenotype consisting of ADHD and conduct disorder, and (3) to present how we clinically managed symptoms and achieved improvement of the behavioral symptoms.

## Case presentation

An 11-year-old boy was referred to the inpatient unit of the University Department of Child Psychiatry, at the “Aghia Sophia” Children’s Hospital, Athens, Greece, for further diagnostic evaluation and treatment by the pediatric liaison team on call. He was socially isolated at school and in the rural community where he lived. He had behavioral difficulties at home and difficulties in adhering to the boundaries set by the parents. His mother labeled him as a ‘troublemaker’ and he was oppositional at school with inappropriate behavior. He was frequently interfering with teaching in the classroom. Although he wanted to socialize with other children, he was clumsy and aggressive in his attempts to initiate contact. Teachers and other children’s’ parent’s complaints objectified the presence of behavioral problems. His behavior was described as aggressive and violent. He had odd interests. His play and his reactions were often inappropriate and fear provoking to others, i.e. he performed animal amputations, made and collected poisons, destroyed objects, and set fires.

### Early development and family history

The patient was born at full term from non-consanguineous parents with a birth weight of 3750 g. He was treated with phototherapy for jaundice and had no further neonatal complications. He was not breast fed, was a quiet baby and in the first 3 months of his life his mother became worried as he was unresponsive to cuddles and hugs. He also never cried. He uttered his first words at 12 months of age, he spoke his first sentence at 2, 5 years of age and at 3, 5 years of age he was fully conversational. The patient’s first three years of life were spent under maternal care until he attended day-care starting at the age of 3.5 years. There, he presented with separation anxiety. Also, he did not spontaneously approach other children, he was very active and he would only take part in activities that interested him. Currently, he attends primary school. He is more sociable, but still he approaches his schoolmates on his own terms. He has severe learning disabilities (especially in reading and writing) and requires extra teaching help. He has no friends and, on occasions, he has been victimized by bullying at school and in the community. He suffers from primary nocturnal enuresis and occasional fecal soiling by day. The patient has two female siblings, an 18 year-old one, currently a university student and recently diagnosed with bipolar disorder, and a 10 year-old one, currently in primary school. His father is 44 years of age and describes having had a difficult childhood; he is characterized by the family as indifferent to the children’s problems and verbally violent towards his wife and son, but less so to his daughters. The mother is 41 years old, and describes herself as having a close relationship with her children and mentioned that she usually covers up for the patient’s difficulties and makes excuses for his violent outbursts. The maternal grandmother suffers from depression and has a history of attempted suicide. She is currently using antidepressant medication. There are also thyroid problems in the two female siblings, the mother and her two sisters; heart problems in four of mother’s siblings and the maternal grandmother; cervical/ovarian cancer in the paternal grandmother; and lung cancer of paternal grandfather. One of the maternal sisters has a history of cervical cancer, heart problems, diabetes mellitus, and psychiatric problems (probably depression; she is currently using medication). Some of the patient’s relatives are also obese or overweight: his mother, his younger sister, maternal grandmother and two of maternal sisters.

### Physical/psychiatric/psychological/occupational therapy examination

The patient was an overweight pre-adolescent of tall stature (height: 167 cm, weight: 73 kg*, BMI: 26.25), as are his older sister, mother, and maternal grandparents. There are no apparent dysmorphias present in the patient, nor in his siblings and parents. He was likeable with elements of oddness during the clinical contact, as well as in the way he walked and moved in the clinic. His medical history includes coloboma of the iris with almost total vision loss of the left eye and myopia and astigmatism of the right eye. He was interviewed by using the semi-structured psychiatric interview K-SADS-PL, and received the diagnosis of both Conduct and Attention Deficit Hyperactivity Disorders. On Children’s Global Assessment Scale (CGAS), he scored 41–50 (moderate effect on functionality). On the Child Behavior Checklist (CBCL), he scored in the clinical range on externalizing (oppositionality, aggression, rule violation, and conduct problems), internalizing (anxiety, depression), and social and attention problems. On the Youth Self-Report (YSR), he scored in the clinical range, as he did on the CBCL. On the ADHD Rating Scale-5 for children and adolescents, the ratings of both parents and teachers were indicative of a combined type ADHD symptoms (inattention and hyperactivity/impulsiveness). The Autism Diagnostic Interview-Revised (ADI-R) was also performed and he scored above the cut-off on restricted, repetitive and stereotype behaviors and interests. In all other ADI-R subscales, the ratings were below the cut-offs for ASD. The psychiatric diagnosis, according to DSM-IV-TR [19] was conduct disorder (childhood onset, 312.81) and ADHD (combined type, 314.01). On axis IV, there were problems with the primary care taking group (lack of support by family, family quarrels), academic problems/difficulties, and conflicts with peers and teachers. The routine laboratory chemistry tests and the structural brain-MRI were normal.

His overall IQ score, as measured by WISC-III-R, was 75, but his profile was considered discordant: his performance IQ was 22 units superior to his verbal IQ, with heterogeneous performance. He had severe difficulties in abstract thinking, verbal conceptualization, and executive functioning while he scored better in visual construct and visual spatial reasoning. The projective tests (H.T.P., C.A.T.) depicted limited emotional intelligence, difficulty in processing things that bothered him, loneliness and rage which he was trying to repress. He needs relationships but doesn’t know how to associate with people and has many projections around aggression and loss.

On the Behavior Observation Guide (BOSS), he had deficits in communication, management of money, educational activities, play, social participation and motor activities (bilateral body coordination, balance, static control and fine activity). On the Occupational Therapy Practice Framework Domain and Process, he demonstrated difficulties in sustaining attention, organizing tasks, remaining seated, and impulsive behaviors; his main difficulty was in visual motor coordination and skills. As a result, on the ABC-2-Movement Assessment Battery for children-2, he scored on the 9th percentile. On the Developmental Test of Visual Perception-2, he performed in most scales below average to normal with the exception of visual motor completion with scores showing significant pathology. The difficulties described above may well have contributed to his reading and writing difficulties. On the Vineland Adaptive Behavior Scales, he performed poor on written and verbal, as well as on community and domestic everyday activities. In general, he presents with mild difficulties in adaptive behavior and moderate maladaptive behaviors.

### Patient’s progress report

During his stay (for 2½ months) in the inpatient unit, the patient underwent psychiatric and pediatric assessments plus occupational therapy. He took part in the unit’s psycho-educational activities and was started on risperidone, 2 mg daily. Risperidone was preferred over an anti-ADHD agent because his behavioral problems prevailed and thus were the main target of treatment. In addition, his behavioral problems had undoubtedly influenced his functionality and mainly his relations with parents, siblings, peers, teachers and others. Risperidone was also preferred over other atypical antipsychotics for its safe profile and fewer side effects. Family meetings were held regularly, and parental and family support along with psycho-education were the main goals. He was aided in recognizing his own emotions and conveying them to others as well as in learning how to recognize the emotions of others and to become aware of the consequences of his actions. Improvement was made in rule setting and boundary adherence. Since his discharge, he received regular psychiatric follow up, continues with the medication and the occupational therapy. Supportive and advisory work is done with the parents. Marked improvement in general has been noticed regarding his social behavior and behavior during activity as described by all concerned. Occasional anger outbursts of smaller intensity and frequency have been reported, but seem more manageable by the child with the support of his mother and teachers.

## Genetic methods

### Isolation of genomic DNA

Genomic DNA was extracted from peripheral blood lymphocytes using standard procedures. The quality and quantity of the DNA samples were determined using the NanoDrop ND-1000 UV–Vis spectrophotometer.

### Cytogenetic analysis

Chromosomal analyses of the patient were performed from peripheral blood samples by conventional G-banding techniques according to the standard protocol. Fifty metaphases, with 550-band resolution (>5 Mb), were analyzed.

### Array comparative genomic hybridization (aCGH)

High resolution aCGH analysis was performed on the DNA sample from the patient. Agilent Human Genome 4X180 K CGH + SNP microarrays with an average spatial resolution of 12 kb was used in the study (Agilent Technologies, Santa Clara, CA). The methodology used for aCGH and analysis of the resulting data was as previously described [20, 21], with the exception of the latest version of CytoGenomics 3.0 software (Agilent Technologies, Santa Clara, CA) being used for both feature extraction and data analysis. For the location of genes in the deleted or duplicated genomic segments the UCSC (http://genome.ucsc.edu/) and the Database of Genomic Variants (http://projects.tcag.ca/variation/; human genome build 19) were used.

Informed consent for genetic testing was obtained from the parents.

## Genetic results

**Fig. 1:**
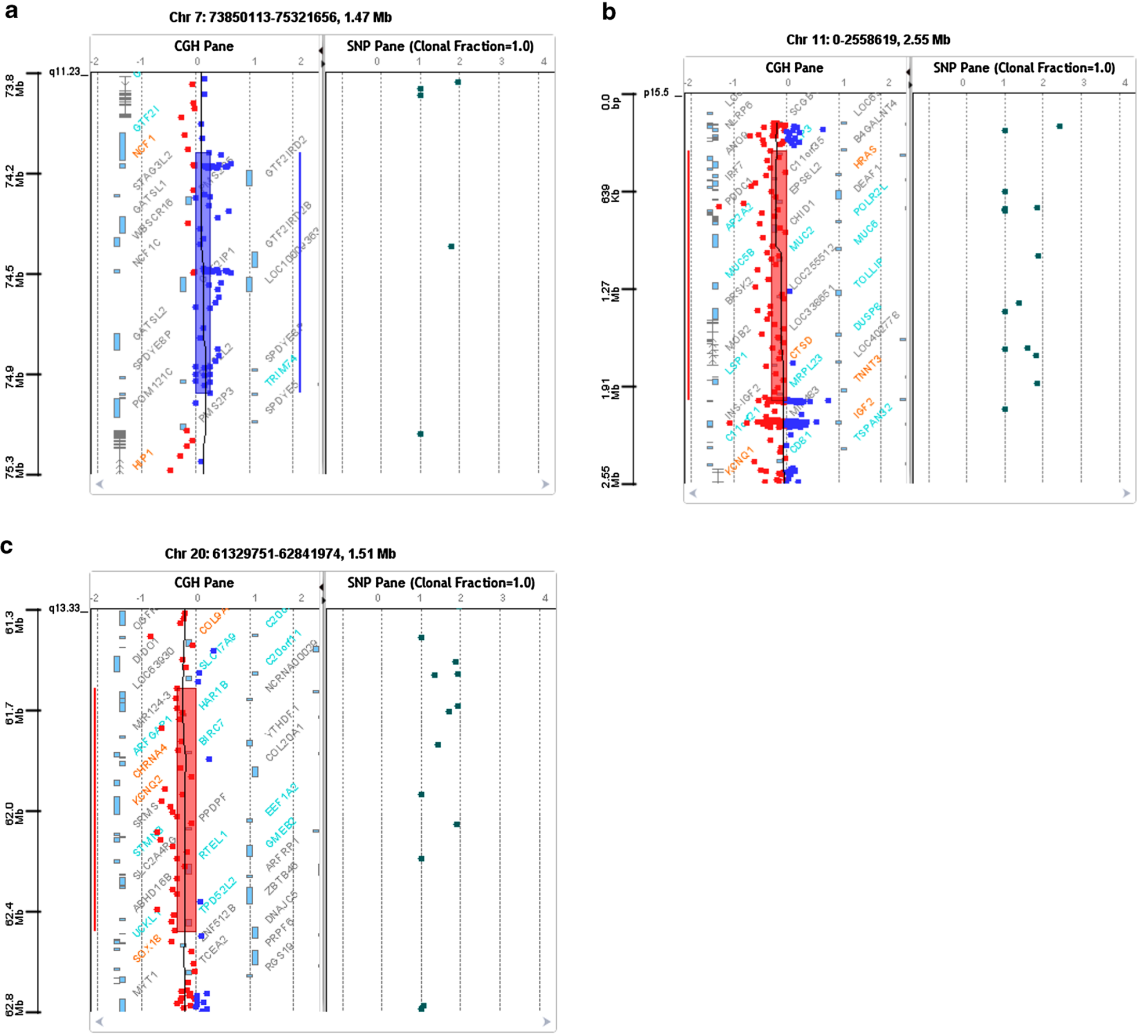
Array comparative genomic hybridization (Agilent 4X180 K) **a** image of the 7q11.23 (882.9 kb) duplicated region, **b** image of the 11p15.5 (1.6 Mb) deletion, **c** 20q13.33 (907.3 Kb) deletion, showing the genes in the specific chromosomal regions (http://genome.ucsc.edu/; GRCh37/hg19)

Conventional G-banding karyotyping revealed 47,XYY karyotype. aCGH testing revealed three additional chromosomal micro-aberrations: an 882.9 Kb duplication on the long arm of chromosome 7 (7q11.23), a 1.6 Mb deletion on the short arm of chromosome 11 (11p15.5), and a 907.3 Kb deletion on the long arm of chromosome 20 (20q13.33) (Table 1, Fig. 1a–c).

**Table 1:** Additional micro-aberrations of the 47,XYY proband

Nomenclature according to ISCN	Size	Important genes
7q11.23 (74,144,422-75,027,348)x3	882.9 Kb	*GTF2I, NCF1, GTF2IRD2, STAG3L2, PMS2P5, GATSL1, WBSCR16, GTF2IRD2B, NCF1C, LOC100093631, GTF2IP1, GATSL2, SPDYE8P, PMS2L2, STAG3L1, TRIM74, TRIM73*
11p15.5 (383,89-2014,937)x1	1.6 Mb	*PKP3, SIGIRR, ANO9, PTDSS2, RNH1, HRAS, LRRC56, C11orf35, RASSF7, MIR210, LOC143666, PHRF1, IRF7, CDHR5, SCT,* ***DRD4*** *, DEAF1, TMEM80, EPS8L2, TALDO1, PDDC1, CEND1, SLC25A22, LRDD, RPLP2, SNORA52, PNPLA2, EFCAB4A, CD151, POLR2L, TSPAN4, CHID1, AP2A2, MUC6, MUC2, MUC5B, TOLLIP, LOC255512, BRSK2, MOB2, DUSP8, LOC338651, KRTAP5*-*1, KRTAP5*-*2, KRTAP5*-*3, KRTAP5*-*4, KRTAP5*-*5, FAM99A, FAM99B, KRTAP5*-*6, LOC402778, CTSD, SYT8, TNNI2, LSP1, MIR4298, TNNT3, MRPL23, LOC100133545*
20q13.33 (61,632,196-62,539,530)x1	907.3 Kb	*BHLHE23, LOC63930, NCRNA00029, LOC100144597, HAR1B* ***, HAR1A*** *, MIR124*-*3, YTHDF1, BIRC7, MIR3196, NKAIN4, FLJ16779, ARFGAP1, MIR4326, COL20A1,* ***CHRNA4, KCNQ2*** *, EEF1A2, PPDPF, PTK6, SRMS, C20orf195, PRIC285, GMEB2, STMN3, RTEL1, RTEL1*-*TNFRSF6B, TNFRSF6B, ARFRP1, ZGPAT, LIME1, SLC2A4RG, ZBTB46, ABHD16B, TPD52L2, DNAJC5*

(UCSC Genome Browser, human genome build 19)

## Conclusions

47,XYY syndrome is associated with various conditions including conduct problems and ADHD, a finding that applies in the case presented here. Childhood ADHD is strongly associated with a significantly increased risk of comorbid conduct disorder, personality disorder, and substance-related disorders. There is an association between ADHD and the likelihood of having an internalizing or externalizing disorder [22]. Association between sociodemographic factors, high social deprivation, poverty and increased risk of externalizing and internalizing disorders is also observed and the strongest association is that between moderately deprived neighborhoods and ADHD [23].

In the case of this boy, apart from the additional Y chromosome, three additional copy number variants (CNVs) were detected by aCGH. The duplication at 7q11.23 is reciprocal to the deletion associated with Williams–Beuren syndrome. Both deletions and duplications at this locus are associated with behavioral problems (anxiety disorders, ADHD, ASD), facial dysmorphisms, developmental coordination disorders, speech sound disorders and medical problems, and MRI structural abnormalities [14, 18]. The 11p15.5 microdeletion contains amongst others the *DRD4* gene responsible for regulating many neurological functions related to psychiatric disorders, developmental delays, as well as to neuroleptic response [24]. However, the molecular-pharmacological effect of the loss of one entire copy of the gene in this specific and other polymorphisms remains to be elucidated. The deletion at 20q13.33 contains three genes (*HAR1A*, *CHRNA4*, and *KCNQ2*) known to be associated with clinical phenotypes. The *HAR1A* gene is considered an accelerated evolution gene for human brain development with molecular defects of the gene leading to psychotic and disruptive disorders [25]; the genes *CHRNA4* and *KCNQ2* also deleted have been associated with ASD and epilepsy [26, 27].

The 47,XYY syndrome has traditionally been associated with increased behavioral problems and to criminal behavior. We also know that 47,XYY is associated with ADHD and ASD symptoms, developmental delays and learning disabilities [3, 7, 10, 28]. It is well known that boys with co-occurring ADHD and conduct problems are identified as being at high risk of lifelong trajectories of delinquent behavior and antisocial personality [29], and excess mortality mainly driven by deaths from unnatural causes, especially accidents [30]. Margari et al. [31] found that juvenile offenders who had committed crimes against people showed more ADHD symptoms and conduct problems than adolescents who had committed property crimes and alcohol-drug-related crimes.

Nevertheless, the relevant studies have different methodological limitations [32]. A recent Danish cohort study showed that convictions were significantly increased among men with 47,XYY syndrome compared to controls in all crime types, except for drug-related crimes and traffic offenses. Importantly, when adjusting for social economic status (SES), the total conviction rates were similar to controls, which means that the increased risk of convictions may be partly or fully explained by poor SES [33].

High levels of childhood inattention and impulsivity increase the likelihood that parents will respond with harsh discipline, inappropriate withdrawal from the child, or inconsistent parenting [34, 35], a finding that also applies in our case. The combination of various interventions adapted to the specific patient’s developmental level i.e. milieu-therapy, during his inpatient stay, positive reinforcement and rewarding techniques, drug administration, psycho-education and support of the patient and his family all may have contributed to his improvement; special attention was given to the patient-parent interaction as his disturbed behavior, aggressiveness and impulsiveness had led the parents to respond as described above. Nevertheless, it is not easy to determine which treatment element was most effective. Low self-esteem, impulsivity and emotional immaturity due to 47,XYY syndrome often leads to self-harming and suicidal behaviors while conduct disorder is associated with major depression [36]. Learning difficulties due to ADHD and 47,XYY syndrome lead children to be at increased risk for social isolation and school dropout. These findings, in combination with rigid thinking and impulsiveness [37], underline the need for early psycho-social-educational intervention and parental training [8].

Genetic diagnostic testing can be very informative in the diagnostic process and should be applied also to patients in Psychiatry in case of complex clinical presentation when multiple organ systems are affected. Multisystem anomalies are indicative of potentially identifiably genetic aberrations. Many of the currently known microdeletion and microduplication syndromes have been associated to somatic symptoms and dysmorphological features such as is the case for 22q11.2 microdeletion syndrome [38, 39]. These dysmorphisms have found to be enriched in the psychiatric patient population [40]. In the case presented in this paper, iris coloboma was the symptom that led us to perform genetic testing with aCGH. Known pathogenic microdeletions and duplications have highly variable phenotypes ranging from only minor dysmorphisms to the face or hands to obesity, speech difficulties, macrocephaly, and intellectual disability [41].

In the case presented here, the history of abuse by the parents, the disrupted family relations, the bullying by his peers, the educational difficulties, and the poor SES could be identified as additional risk factors relating to bad prognosis. As good prognostic factors we can identify the ending of the abuse after intervention, the child’s encouragement and support from parents and teachers and the improvement of parental relations as results of parent training and family support by mental health professionals. Taken together, it appears that also in case of psychiatric patients presenting with complex genetic aberrations and additional psychosocial problems, traditional psychiatric and psychological approaches can lead to decrease of symptoms and improved functioning.

## Abbreviations

CD: conduct disorder
ADHD: attention deficit hyperactivity disorder
CGH: comparative genomic hybridization
ASD: autism spectrum disorders
WBS: Williams–Beuren Syndrome
CGAS: children’s global assessment scale
CBCL: child behavior checklist
YSR: youth self-report
ADI-R: autism diagnostic interview-revised
BOSS: behavior observation guide
aCGH: array comparative genomic hybridization
